# The Effect of Three-Month Vitamin D Supplementation on the Levels of Homocysteine Metabolism Markers and Inflammatory Cytokines in Sera of Psoriatic Patients

**DOI:** 10.3390/biom11121865

**Published:** 2021-12-11

**Authors:** Alma Prtina, Nela Rašeta Simović, Tatjana Milivojac, Milorad Vujnić, Milkica Grabež, Dragan Djuric, Miloš P. Stojiljković, Valentina Soldat Stanković, Miodrag J. Čolić, Ranko Škrbić

**Affiliations:** 1 Center for Biomedical Research, Department of Pathophysiology, Faculty of Medicine, University of Banja Luka, 78000 Banja Luka, Bosnia and Herzegovina; alma.prtina@med.unibl.org (A.P.); nela.raseta@med.unibl.org (N.R.S.); tatjana.milivojac@med.unibl.org (T.M.); milorad.vujnic@med.unibl.org (M.V.); 2 Department of Preventive Medicine, Faculty of Medicine, University of Banja Luka, 78000 Banja Luka, Bosnia and Herzegovina; milkica.grabez@med.unibl.org; 3 Institute of Medical Physiology “Richard Burian”, Faculty of Medicine, University of Belgrade, 11000 Belgrade, Serbia; dr_djuric@yahoo.com; 4 Faculty of Medicine, University of Banja Luka, 78000 Banja Luka, Bosnia and Herzegovina; 5 Department of Pharmacology, Toxicology and Clinical Pharmacology, Faculty of Medicine, University of Banja Luka, 78000 Banja Luka, Bosnia and Herzegovina; milos.stojiljkovic@med.unibl.org; 6 Internal Medicine Clinic, University Clinical Center of the Republic of Srpska, Faculty of Medicine, University of Banja Luka, 78000 Banja Luka, Bosnia and Herzegovina; valentina.soldat@kc-bl.com; 7 Medical Faculty Foča, University of East Sarajevo, 73300 Foča, Bosnia and Herzegovina; miocolic@gmail.com

**Keywords:** psoriasis, vitamin D supplementation, homocysteine, cytokine

## Abstract

Psoriasis is an autoimmune and inflammatory skin disease. Psoriatic patients express higher levels of plasma homocysteine (Hcy) concentration and pro-inflammatory mediators than healthy people; this is frequently associated with vitamin D deficiency. The aim of this clinical study was to investigate the effects of high doses of vitamin D supplementation on the parameters of Hcy metabolism and cytokines in sera of psoriatic patients. This prospective study was conducted on 40 psoriatic patients who had the vitamin D deficiency. All patients received vitamin D 5000 IU/day for three months. Clinical and biochemical measurements were taken at baseline and at follow up (3 months). The results showed that the severity of clinical features, measured by the psoriasis area severity index (PASI) score, were considerably improved in patients after vitamin D supplementation. After vitamin D supplementation, most of the patients (*n* = 25 or 62.5%) had mild clinical form (*p* < 0.001). After twelve weeks of intervention period, there were significant increases in vitamin D and B_12_ serum levels in comparison to the levels that had been measured at the beginning of the study (56.77 ± 14.66 nmol/L and 301.08 ± 95.02 pg/mL vs. 103.85 ± 32.20 nmol/L and 362.81 ± 118.56 pg/mL, respectively; *p* < 0.001). Moreover, serum levels of Hcy and folate were significantly lower at the end of the study in comparison with the initial levels (12.45 ± 1.92 µmol/L and 8.01 ± 3.88 mg/mL vs. 10.38 ± 1.66 µmol/L and 6.27 ± 2.60 mg/mL, respectively). High doses of vitamin D supplementation led to a significant decrease in pro-inflammatory cytokines (IFN-ɤ, TNF-α, IL-1β, IL-6, IL-8, and IL-17) and high-sensitivity C-reactive protein (hsCRP), whereas the production of anti-inflammatory cytokines (IL-10, IL-5) was up-regulated. In conclusion, supplementation with high doses of vitamin D could be one of the possible preventive and therapeutic measures to reduce systemic inflammation in psoriatic patients.

## 1. Introduction

Contemporary research suggests that psoriasis is a multifactorial disease mediated by immune mechanisms with a clear genetic predisposition. Clinically, psoriasis is usually manifested as plaque psoriasis [1]. Histologically, plaque psoriasis is characterized by hyperproliferation and differentiation of epidermal keratinocytes as well as by inflammatory cell infiltration into skin including neutrophils, dendritic cells, and T lymphocytes [1,2]. Although the pathogenesis of psoriasis has still not been completely illuminated, a large amount of data accumulated to date indicate a complex interaction between epidermal keratinocytes and inflammatory cells. The unknown triggering environmental factors change antimicrobial peptide LL37, melanocytic peptide ADAMTS-like protein 5, and other biomolecules from keratinocytes to act as autoantigens with the potential to induce IL-17A expression by reactive T cells through the IL-23/Th-17 axis. Th17 cytokines, especially IL-17A, IL-17F, and IL-22, through a complex influence on different immune cells of both innate and adaptive immunity as well as on non-immune cells (keratinocytes, fibroblasts, and endothelial cells) promote the typical characteristics of psoriatic skin lesions. In this context, activated keratinocytes orchestrate subsequent immuno-inflammatory responses [3]. Keratinocyte activation implies production of various mediators, such as cAMP, proinflammatory cytokines (IL-1, IL-6, and TNF-α), chemokines (IL-8/CXCL8, CXCL11, and CCL20), IL-17C (a unique keratinocyte cytokine), and S100A proteins that further increase the migration of immune cells into the psoriatic lesion, activate stromal cells in the dermis layer, and induce angiogenesis [4,5]. On the other hand, activated immune cells, which have been accumulated in psoriatic plaques, create a cytokine “milieu” that activate psoriatic keratinocytes [3,4]. In the chronic phase of psoriasis Th1 cells predominate. These cells produce IFN-γ, IL-2, and TNF-α. The key inducer of their differentiation is IL-12, a cytokine dominantly produced by dendritic cells. Besides the dominant IL-23/IL-17 axis, new data have indicated an increased expression of IL-36 cytokines in epidermal cytokines of psoriatic dermal lesions in humans [6,7]. Animal studies conducted in mice have also emphasized the key role of IL-36 in the creation and amplification of psoriatic inflammation, together with IL-23/IL-17 pathway [2,7]. An essential mechanism of psoriasis pathogenesis is the dysfunction of T regulatory cells (Treg), which are involved in immune homeostasis and maintenance of tolerance to prevent autoimmunity [8].

Besides the local inflammatory changes in the skin, psoriatic patients have symptoms of systemic inflammation which are in the bases of various comorbidities that occur in these patients, such as polyarthritis, dyslipidemia, arterial hypertension, insulin resistance and metabolic syndrome, diabetes mellitus, oxidative stress, atherosclerosis, and cardiovascular diseases related to it [9,10,11,12]. Hyperhomocysteinemia (HHcy) is an independent factor associated with cardiovascular diseases (CVD) [13].

The latest studies have shown that psoriatic patients more frequently have HHcy compared to the healthy control group, which might be one of the reasons why there have been more CVDs in psoriatic patients. However, some authors also suggest a possible role of homocysteine (Hcy) in pathogenesis of psoriasis through an increased activation of Th1 and Th17 lymphocytes [14,15,16,17,18].

Hcy is a sulfuric amino acid that is part of a complex methionine–homocysteine–methionine cycle in which vitamin B_12_ and folic acid, as coenzymes, play a crucial role. High blood levels of Hcy in psoriatic patients could be influenced by folate deficiency as a consequence of their increased utilization by keratinocytes, which rapidly proliferate in psoriasis. Folate deficiency might be explained by their decreased absorption in the small intestine due to microscopic inflammatory changes of the small intestinal mucosa, which have been found in these patients. Furthermore, patients with psoriasis have lower blood levels of vitamin B_12_ in comparison with the healthy control group, which might contribute to the occurrence of HHcy [16,17].

Many studies have indicated that patients with psoriasis have low vitamin D blood level. Receptors for vitamin D (VDR) are located in various tissues and organs; therefore, it is not a surprise that vitamin D deficiency has been connected to a considerable number of illnesses and pathological conditions such as obesity, insulin resistance, arterial hypertension, and dyslipidemia [19,20]. Vitamin D supplementation has been used in psoriasis for years and the mechanism of its efficiency includes binding to VDR of epidermal keratinocytes, inducing their differentiation. At the same time, it inhibits their proliferation and thus breaks IL-23/IL-17 inflammatory axis. Vitamin D also exerts an immunomodulatory effect on monocytes, macrophages, T lymphocytes, and dendritic cells [2,7]. The latest data show an inverse relationship between vitamin D and Hcy blood levels in the overall population, as well as in a series of pathological conditions. Studies have shown that vitamin D directly affects the transcription of genes involved in Hcy metabolism through its receptor which functions as a ligand. Patients with psoriasis often have low blood levels of vitamin D, vitamin B_12_ and folic acid, associated with high blood levels of Hcy, which synergistically increases the risks for CVD in multiple ways [21,22].

Based on a literature search, there is a limited number of clinical studies that provide insight on this topic. Therefore, the aim of this study was to examine the effects of high doses vitamin D supplementation on the levels of homocysteine, vitamin B_12_, and folate, pro-inflammatory and anti-inflammatory cytokines in sera of patients with plaque psoriasis, as well as the interconnection of these biomarkers and their correlation with the clinical response following the therapy.

## 2. Materials and Methods

### 2.1. Ethical Issues

The study was approved by the Ethics Committee of the University Clinical Centre of the Republic of Srpska, Banja Luka, Bosnia and Herzegovina (Permission number: 01-9-740.2/16). The patients were provided with detailed information about the study, and prior to enrolment in the study an informed consent was signed by each participant.

### 2.2. Study Design

The study was conducted as a prospective clinical study in chronic plaque psoriatic patients from June to October 2018. Patient’s data were collected at the Skin and Venereal Diseases Clinic of the University Clinical Centre of the Republic of Srpska. The overall sample consists of 40 patients with chronic plaque psoriasis and the inclusion criteria were: patients above 18 years of age, vitamin D serum concentration < 75 nmol/L, serum calcium concentration within the optimal range (1.9–2.6 mmol/L), lack of use of any systemic anti-psoriatic or local therapy, including phototherapy and topical medication of vitamin D for at least three months, as well as lack of use of vitamin D supplements for at least three months before they entered the study. The study participants had no co-morbidities and therefore were not treated with any medications.

After this, all patients were instructed to take vitamin D 5000 IU once daily for three months. The participants were given a precise number of capsules needed for three-month therapy and their compliance was individually controlled on a monthly basis during clinical check-ups.

### 2.3. Clinical and Demographic Characteristics

All psoriatic patients were diagnosed in accordance with the typical clinical features such as occurrence of characteristic and clearly limited erythematous plaques with non-adherent whitish desquamation, which had been confirmed with pathohistological (PH) examination. Anthropometric parameters such as weight, height, and body mass index (BMI), as well as the demographic patient’s characteristics, were collected.

Psoriasis Area Severity Index (PASI) score was used in order to assess activity of the disease [23]. This activity estimates the skin area affected by changes (erythema, infiltration, and quantity of desquamation). Based on PASI score values, patients were categorized into three groups: mild form (low values of PASI ˂ 10); moderate form (moderate values of PASI 10–20); severe form of the disease (high values of PASI ˃ 20).

### 2.4. Biochemical Analyses

Blood samples were taken from all study participants the for the following biochemical analyses: Hcy, vitamin B_12_, folate, high-sensitivity C-reactive protein (hsCRP), cytokines, INF-ɤ, TNF-α, IL-1β, IL-6, IL-8, IL-17, IL-10, and IL-5. These parameters were measured at the beginning of the study and three months after taking vitamin D supplementation. Serum calcium level was monitored monthly.

### 2.5. Measuring Techniques

Total electrochemiluminscence immunoassay on Cobas e411 (Roche Diagnostics) was used to determine the vitamin D serum concentrations. The following reference values were used: <50 nmol/L–vitamin D deficiency; <75 nmol/L–vitamin D insufficiency; and ≥75 nmol/L–vitamin D optimal levels.

The concentration of serum Hcy was determined using an enzymatic cycling assay by using the Cobas 6000 analyzer (Roche Diagnostics GmbH, Mannheim, Germany). Reference values for Hcy were 0–12 µmol/L.

Serum folate was measured using electrochemiluminescence immunoassay (Roche Diagnostics, Mannheim, Germany). Reference values for folate were 3.89–26.8 mg/mL. Vitamin B_12_ concentration in serum was measured using chemiluminiscence assay (ADVIA Centaur XP, Siemens Healthineers, Malvern, PA, USA, equipment has been sourced from USA). Reference values for vitamin B_12_ were 211–911 pg/mL.

The cytokine concentration in serum samples were measured by flow cytometry with Aimplex Human Th1/Th2 10-plex (Applied biosistems by life technologies, Thermo Fisher Scientific, Waltham, MA, USA). Serum level of IL-17 was determined with a high sensitive human IL-17 ELISA kit (R&D Systems, Minneapolis, MN, USA) according to the manufacture’s instruction.

### 2.6. Statistical Analyses

Statistical analyses were performed using IBM SPSS 20 (Chicago, IL, USA). For each variable, Shapiro–Wilk’s test was used to assess normality of distribution. Data obtained were expressed as mean ± standard deviation for continuous variables or number (%) for the categorical variables. For comparisons between groups at baseline Chi-square test and the One-way analysis of variance (ANOVA) were used. For comparisons within groups between two time points, a paired *t* test (for normally distributed data) or Wilcoxon’s matched pairs test (for data without normal distribution) were used. The correlation was found with the help of Pearson’s correlation coefficient. A value of *p* < 0.05 was considered as statistically significant. A sample size of 40 was required to detect an effect of treatment, at 80% statistical power and an α-level of 0.05.

## 3. Results

### 3.1. Demographic Data in Psoriatic Patients

The study was conducted as a prospective intervention study in 40 psoriatic patients of both sex (22 females, 55%) and average age of 47.13 ± 15.10 years. According to the severity of the disease measured with PASI score, patients were divided into three groups (mild, moderate, and severe forms). There were no significant differences related to age, sex, and smoking status, duration of the disease, family history of psoriasis, and the previous duration of the disease in study groups. The patients with severe forms of psoriasis also had higher levels of BMI in comparison with mild and moderate forms (27.97 ± 1.35 kg/m^2^ vs. 25.72 ± 2.66 kg/m^2^ and 25.79 ± 2.89 kg/m^2^). However, this discrepancy did not reach the level of statistical significance (*p* = 0.051) (Table 1).

### 3.2. The Effect of Vitamin D Supplementation on Disease Severity in Psoriatic Patients

At the beginning of the intervention period, there was no significant difference in groups according to the severity of clinical features measured by PASI score. Supplementation of high doses of vitamin D for 12 weeks significantly improved the clinical features in patients with severe and moderate form of psoriasis. After vitamin D supplementation, most of the patients (*n* = 25 or 62.5%) had mild clinical form (*p* < 0.001) (Table 2).

### 3.3. Serum Levels of Vitamin D, Homocysteine, VITAMIN B_12_, and Folate in Psoriatic Patients

**Figure 1:**
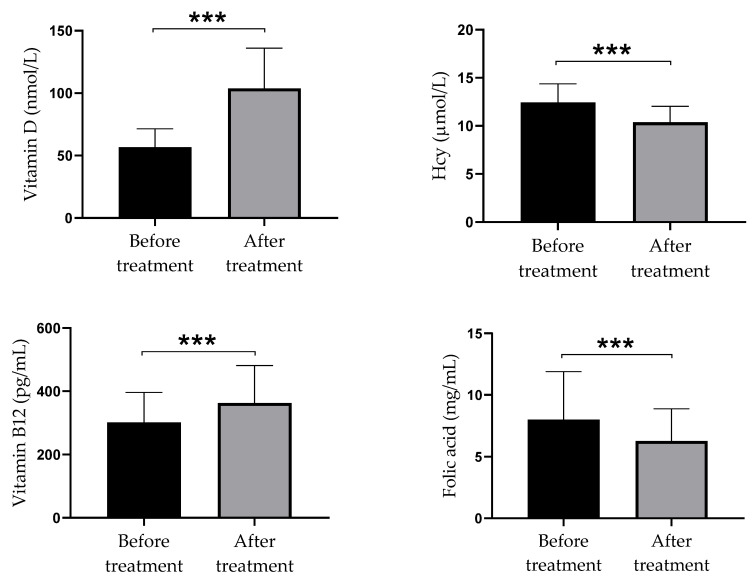
Serum levels of vitamin D, Hcy, vitamin B_12_, and folate in psoriatic patients at the baseline and after twelve weeks of high-dose of vitamin D supplementation. Values are presented as mean ± standard deviation (*n* = 40); paired samples *t*-test was used to compare mean values before and after treatment, the statistical significance is shown *** *p*  <  0.001). Abbreviation: Hcy = homocysteine.

After twelve weeks of intervention period, there were statistically significant increases in vitamin D and B_12_ serum levels in comparison to the levels that had been measured at the beginning of the study (56.77 ± 14.66 nmol/L and 301.08 ± 95.02 pg/mL vs. 103.85 ± 32.20 nmol/L and 362.81 ± 118.56 pg/mL, respectively; *p* < 0.001). Moreover, serum levels of Hcy and folate were significantly lower at the end of the study in comparison with the initial levels (12.45 ± 1.92 µmol/L and 8.01 ± 3.88 mg/mL vs. 10.38 ± 1.66 µmol/L and 6.27 ± 2.60 mg/mL, respectively) (Figure 1).

### 3.4. Differences in Serum Levels of Vitamin D, Homocysteine, Vitamin B_12_, and Folate between Psoriatic Patients before and after Vitamin D Supplementation

Table 3 shows categorical values for vitamin D, homocysteine, vitamin B_12_, and folate at the beginning and at the end of the intervention period. As one of the criteria for participation in the study was low serum values of vitamin D (vitamin D < 75 nmol/L), it was detected that 80% of patients (*n* = 32) achieved optimal levels of vitamin D after the intervention (*p* < 0.001). A significant improvement in Hcy values was also detected.

At the beginning of the study, 37.5% of patients had desirable levels of Hcy, while after supplementation with high doses of vitamin D, 82.5% of them reached optimal levels (*p* < 0.001). Most patients, about 90%, had optimal levels of vitamin B_12_ and folate at the beginning of the study, therefore these categories did not show significant changes (Table 3).

### 3.5. The Concentration of Pro-Inflammatory and Anti-Inflammatory Parameters in Serum of Psoriatic Patients before and after Supplementation with Vitamin D

**Figure 2:**
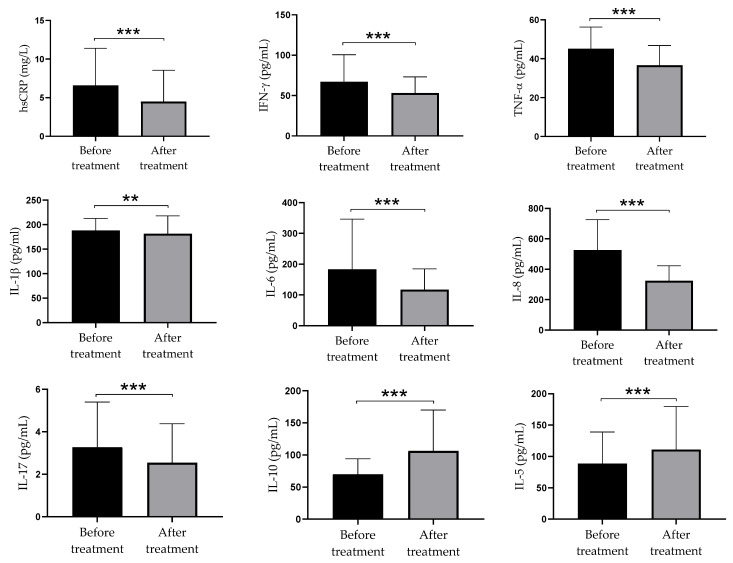
The concentration of pro-inflammatory and anti-inflammatory parameters in serum of psoriatic patients before and after twelve weeks of supplementation with high doses of vitamin D. Values are presented as mean ± standard deviation (*n* = 40). Wilcoxon Signed Rank Test was used to compare mean values before and after treatment; the statistical significance is shown (** *p*  <  0.01, *** *p*  <  0.001). Abbreviation: hsCRP = high-sensitivity C-reactive protein.

High doses of vitamin D supplementation led to a significant decrease in production of pro-inflammatory parameters (hsCRP, IFN-γ, TNF-α, IL-1β, IL-6, IL-8, and IL-17) and increase in production of anti-inflammatory parameters (IL-10 and IL-5). Serum levels of pro-inflammatory and anti-inflammatory cytokines are presented in Figure 2.

### 3.6. Relationship between Serum Hcy and Pro-Inflammatory and Anti-Inflammatory Parameters in Serum of Psoriatic Patients before and after Supplementation with Vitamin D

At the beginning of the intervention period the correlation between Hcy and pro-inflammatory and anti-inflammatory parameters was not significant. However, after twelve weeks of intervention period, the negative moderate statistically significant correlation between Hcy and pro-inflammatory cytokines IL-1β (r= −0.338; *p* = 0.035) and Hcy with IL-8 (r = −0.374; *p* = 0.019) was observed, which means that high values of Hcy after twelve weeks of intervention period was followed by lower concentrations of IL-1β and IL-8 (Table 4).

## 4. Discussion

Vitamin D deficiency is a condition associated with the development of several chronic diseases, including CVD and metabolic diseases. There is also a significant correlation between vitamin D deficiency and mortality for major cardiovascular events such as myocardial infarction, heart failure, atrial fibrillation, sudden cardiac death, and peripheral vascular disease [24]. Psoriasis is a chronic inflammatory skin disease, caused by complex interactions between environmental factors and the immune system in persons with specific genetic background. It is associated with vitamin D deficiency and various abnormalities in cytokine network [17]. Psoriasis is very often accompanied by dysregulation of Hcy metabolism. As there is strong evidence that Hcy plays a significant role in immune-inflammatory pathogenesis of the disease [1,17], the aim of our study was to investigate whether and how these parameters in sera of psoriatic patients change after supplementation with high dose of vitamin D.

Our study showed that after three months of vitamin D supplementation, as many as 80% of patients had achieved the optimal vitamin D concentrations and this effect correlated with clinical improvement of skin lesions, as judged by the decreased PASI scores. These results are in accordance with previous findings which were systematized in a very recent review [25]. Such a clinical improvement was expected, as the relationship between vitamin D deficiency and psoriasis is well established [26]. Our first aim was to check how this therapy modifies the levels of Hcy and coenzymes (B_12_ and folic acid) through measured values of folate, involved in Hcy metabolism.

The Hcy blood concentration is elevated in the immune-inflammatory reaction and leads to autoimmune diseases. There are studies that show that different genes in epidermal, blood, and bone marrow cells are more or less methylated in patients with psoriasis compared to the control group. DNA methylation is the leading epigenetic factor thought to be responsible for the pathogenesis of psoriasis, and it has been proven that Hcy and methionine induce methylation of the DNA in patients with psoriasis [9,15,27,28].

In accordance with this, the results of this study showed the prevalence of HHcy in nearly 64% of patients. Results of other authors seem to be incoherent. Most studies showed the presence of Hhcy in patients with psoriasis, which is consistent with the results of this study [11,17]. On the contrary, Cakmak et al. did not find any significant differences in the concentration of Hcy, folic acid and vitamin B_12_ between the patients with psoriasis and the healthy control group [29]. The study by Uslu et al. found no evidence of differences in levels of Hcy, folic acid, and vitamins B_12_ and B_6_ in plasma of psoriasis patients and controls; the reasons for these discrepancies have not yet been explained [30].

In our study, we found a statistically significant increase in serum vitamin B_12_ concentrations three months after treatment with high doses of vitamin D supplementation, but serum Hcy and folate values were significantly lower than at the baseline.

The literature data related to the effect of vitamin D therapy on Hcy metabolism in patients with psoriasis are not consistent. Several studies showed a significant inverse correlation between HHcy and vitamin B_12_ blood levels but did not find any correlation between levels of Hcy and folate. The possible explanation of low blood level of folate in patients with psoriasis lies in rapid keratinocyte proliferation, which leads to the increased folate consumption. A possible cause may also be found in micro-inflammatory changes in the colon mucosa in psoriatic patients, which lead to reduced absorption of folate [16,18,28]. Many authors did not succeed in finding any significant correlation between PASI and Hcy, vitamin B_12_, and folate levels. Cakmak et al. [29] found a direct inverse correlation between PASI and Hcy but did not detect any correlation between Hcy and folic acid. However, in other studies, an inverse correlation between folic acid or folate and Hcy were found [9,28,29,30]. The present study shows that serum levels of both Hcy and folate are significantly decreased after vitamin D supplementation. Even though literature data mostly suggest that folate levels are lower in psoriatic patients and folate deficiency can contribute the increased Hcy level [9,28,29,30], the full range of mechanisms by which folate deficiency may contribute the elevation of Hcy level is unclear. One of the major potential factors is that folate is necessary cofactor for the enzyme 5, 10-methyenetetralhydrofolate reductase which mediates conversion of Hcy to methionine [31]. However, our results show a strong negative correlation between folate and Hcy, both before (r = −0.436; *p* = 0.006) and after vitamin D supplementation (r = 0.392; *p* = 0.015) (data not shown). This means that even the levels of folate and HCy were decreased after vitamin D supplementation in psoriatic patients; the decrease at follow up was associated with significantly lower values of folate when compared to Hcy. Although the lower values of folate after vitamin D supplementation were not excepted, the negative correlation between the folate and Hcy levels was nevertheless maintained.

It is well known that the active form of vitamin D regulates the expression of many genes by binding to its VDR receptor, which participates in the transcription of several genes. Additionally, vitamin D regulates genes that encode activities of enzymes involved in Hcy metabolism. The cystathionine beta-synthase has been shown to be the target gene of VDR regulation, making the active form of vitamin D a potential regulator of Hcy metabolism [20,21,22].

A significant part of our study was related to the modulatory role of high doses of vitamin D supplementation on the balance between pro- and anti-inflammatory parameters within a complex cytokine network in psoriasis. It is well documented that psoriasis is followed by a significant production of pro-inflammatory cytokines, not only in the skin lesions but also in systemic circulation [1,2,4]. In a recent meta-analysis of 2876 psoriasis patients, increased serum levels of TNF-α, IFN-γ, IL-2, IL-6, IL-8, IL-18, and IL-22 in comparison with 2237 healthy controls have been documented, whereas no differences were found in the levels of IL-1β, IL-4, IL-10, IL-12, IL-17, IL-21, and IL-23 [31]. It is interesting that IL-17 was up-regulated only in men, although psoriasis in women was accompanied by a lower level of vitamin D than in men. In this context, Priyadarssini et al. showed significant changes in phenotypes of T cells in psoriatic patients, manifested by an increase in the frequency of Th1/ Th17 subsets and a relative decrease in Th2/Treg cells compared to healthy persons [32]. They also found a positive correlation between the percentages of Th1/Th17 cells and PASI scores. The differences between T subset number and produced cytokines in serum partly differ due to different methods used for measurements and their sensitivity. It is especially significant for IL-17 where only ultrasensitive ELISA is applicable. Although we did not use a healthy control group, our findings that high doses of vitamin D led to a significant decrease in pro-inflammatory parameters (hsCRP, IFN-ɤ, TNF-α, IL-1β, IL-6, IL-8, and IL-17) suggest that their levels in systemic circulation in psoriatic patients were elevated. In contrast, the therapy is followed by a significant increase in the levels of IL-5 (a Th2 cytokine) and IL-10 (a key Treg cytokine).

Our findings that vitamin D supplementation decreased the serum level of IL-17 is a very important and an expected phenomenon, bearing in mind the crucial role of the IL-23/IL-17 axis, in both initiation and propagation of skin lesions in psoriasis. Our results are also in accordance with previous findings by other authors showing that application of vitamin D and its analogs on psoriatic lesions significantly decreased the infiltration of Th17 cells in the skin and inhibited their ex vivo expansion [33,34]. The studies investigating serum IL-17 concentrations in psoriasis after systemic vitamin D therapy are very scarce. The down-regulating effect of vitamin D on IL-17 could be explained by its direct inhibition of ROR gamma, a main transcription factor of IL-17, inhibition of IL-23 production by DC, suppression of certain beta-defensins, cathelicidin, and psoriasin in keratynocytes (all biomolecules are involved in the promotion of the Th17 response) [5]. One of the mechanisms involved in the suppression of Th17 cells after vitamin D therapy was mediated through Treg. The inverse correlation between Treg and Th17 is well established and plays a role in many autoimmune diseases. Although there have been conflicting reports in the literature on the role of different types of regulatory cells and anti-inflammatory cytokines in psoriasis, most papers emphasized the key importance of Tregs. It has been shown that serum levels of vitamin D in psoriasis inversely correlated with the number of circulating Treg. It is postulated that vitamin D directly induces differentiation of Treg with immunosuppressive activity mediated by IL-10, including enhanced expression of IL-10 within the psoriatic lesions [8]. However, Treg can be generated from the naive T cells by the influence of dendritic cells after their exposure to vitamin D, which causes these antigen-presenting cells to be tolerogenic [8]. Our finding about increased levels of IL-10 in psoriatic patients after vitamin D therapy is consistent with this concept. IL-10 is able to suppress not only IL-17, but also Th1 and other pro-inflammatory cytokines. This might be one of the mechanisms involved in the down-regulation of pro-inflammatory cytokines after vitamin D therapy. Moreover, direct inhibitory effect of vitamin D on NF-kappa B, a key transcription factor involved in the production of pro-inflammatory cytokines (IL-1, IL-6, TNF-α, IL-8, and IFN-γ) in both T cells and innate immunity cells is of additional significance [5]. It is logical to conclude that lower production of IL-6 has direct effect on decreased production of CRP.

The promotion of Th2 response by vitamin D is also a well-known phenomenon observed both in experimental and clinical studies. The dominant cytokines of Th2 cells are IL-4, IL-5, and IL-13. Of these cytokines, we analyzed IL-5 and found its significant up-regulation after vitamin D therapy. There are several possible mechanisms relevant for up-regulated Th response. The first is related to the suppressed Th1 response, as Th1 and Th2 are mutually antagonistic. Th2 cytokines are also negative regulators of Th17 cell differentiation. In the context of this mutual antagonism, Th2 responses can be also augmented, when IL-12/IL-23 production by dendritic cells is silenced as demonstrated in a murine model of experimental encephalomyelitis [35].

The direct effect of vitamin D on the differentiation of Th2 cells is also possible. The significance of IL-4 in psoriasis is documented through inhibition of IL-1β and IL-6 secretion by epidermal cells from psoriatic lesions [36] and suppression of psoriasis-associated biomolecules in the skin such as IL-8, IL-19, and beta-defensin 2 [36,37].

There is a number of data in the recent literature that indicates the presence of markers of acute and chronic inflammation as well as HHcy in patients with psoriasis. This could be explained in a way that different inflammatory factors not only initiate the sequential inflammatory pathway, including acute and chronic inflammation, but can also cause HHcy due to vitamin deficiency using different pathways. As there are two different inflammatory pathways, one for Hcy production and one for increasing markers of acute and chronic inflammation, the elevated circulating Hcy and elevated markers of acute and chronic inflammation are not always correlated [9,10,18,29]. These results coincide with our findings. At baseline, correlation between Hcy and inflammatory parameters was not significant. It is believed that the presence of markers of inflammation depends on the length of the presence of the inflammatory factors [16,27,38,39,40]. However, after twelve weeks of vitamin D supplementation, a negative correlation between Hcy and pro-inflammatory cytokines IL-1β and IL-8 was observed, which could suggest that higher values of Hcy after intervention period were followed by lower pro-inflammatory immune response. These results could be explained by the regulatory effect of vitamin D supplementation on secretion of IL-1β and IL-8. Zhang et al. showed that addition of vitamin D in cultured normal keratinocytes significantly decreased production of IL-1 and IL-8 in culture supernatants [41]. The authors concluded that vitamin D was effective in terms of regulation of both IL-1 and Il-8 secretion by keratinocytes, which may be a significant contribution to the use of this vitamin and its clinical efficacy against psoriasis [40]. Even though studies in mice have shown that anti-inflammatory cytokine IL-10 administration lowers serum Hcy levels, indicating a negative effect of IL-10 on Hcy [15,39,40], our results did not show a significant correlation between Hcy and IL-10 at baseline or at follow-up.

Deficiency of vitamin B_6_, vitamin B_12_, and folic acid is a common cause of HHcy. While inflammation inclines to promote the cell proliferation at the expense of the elevated vitamin values, HHcy may indicate the presence of inflammation. Moreover, inflammation results in an increase in nitric oxide synthesis, which leads to HHcy binding to vitamin B_12_. The Hcy is associated not only with atherogenesis but also with inflammation and autoimmunity [16,27,38,39,40].

Increasing evidence suggests that HHcy is a risk factor for the development of CVD. It also plays a role in the onset and development of tissue damage. The Hcy and its derivates, depending on their concentration, lead to activation or apoptosis of T cells. In the presence of Hcy, stimulation of mononuclear cells or isolated T cells leads to an increase in the production of type 1 (IL-2, IFN-ɤ, IL-10, and TNF-α) but not type 2 (IL-4 and IL5) cytokines [4,7,42,43]. All these facts suggest that vitamin D therapy, by acting directly or indirectly to decrease the levels of Hcy and pro-inflammatory mediators, could be beneficial in alleviating the symptoms of psoriasis and systemic complications of the disease.

Although the number of patients in this study seems small, it was the result of calculation aimed to assure 80% power of the study. Nevertheless, further randomized, double-blind and placebo-controlled studies, involving larger numbers of psoriatic patients, may contribute to better understanding of the additional role of vitamin D on inflammatory and immuno-modulatory pathway in psoriasis. The lack of the control group of healthy individuals may be perceived as a limitation. However, this study was designed to monitor the effects of vitamin D supplementation on the pre-intervention levels of the parameters studied, which means that each subject acted as his/her own control.

## 5. Conclusions

After three months of vitamin D supplementation, as much as 80% of patients with psoriasis had achieved a significant increase in vitamin D and B_12_ concentrations, while serum Hcy and folate values were significantly lower than at baseline. These changes correlated with the clinical improvement of skin lesions. High doses of vitamin D supplementation led to a significant decrease in the production of pro-inflammatory cytokines, and to increase in the production of anti-inflammatory cytokines. These results suggest that high doses of vitamin D could not only be a therapeutic option, but also one of the possible preventive measures to reduce systemic inflammation in patients with psoriasis.

## Figures and Tables

**Table 1:** Demographic baseline data according to the psoriasis area severity index (PASI) score in psoriatic patients.

Variable		Mild (PASI < 10) *n* = 15	Moderate (PASI 10–19) *n* = 14	Severe (PASI ≥ 20) *n* = 11	Total *n* = 40	*p* Value
Gender						
	Male	5 (33.3)	8 (57.1)	5 (45.5)	18 (45.0)	0.479 ^b^
	Female	10 (66.7)	6 (42.9)	6 (54.5)	22 (55.0)	
Age (year)		49.13 ± 15.14	47.0 ± 18.25	44.55 ± 11.06	47.13 ± 15.10	0.755 ^a^
BMI (kg/m^2^)		25.72 ± 2.66	25.79 ± 2.88	27.97 ± 1.35	26.36 ± 2.60	0.051 ^a^
Disease duration (years)		14.67 ± 11.16	12.79 ± 10.32	14.36 ± 15.20	13.93 ± 11.84	0.908 ^a^
Smoking status						
	Smoker	9 (60.0)	10 (71.4)	8 (72.7)	27 (67.5)	0.760 ^c^
	Nonsmoker	6 (40.0)	4 (28.6)	3 (27.3)	13 (32.5)	
Family history of psoriasis						
	positive	8 (53.3)	7 (50.0)	6 (54.5)	21 (52.5)	0.972 ^b^
	negative	7 (46.7)	7 (50.0)	5 (45.5)	19 (47.5)	
Beginning of disease						
	early	7 (46.7)	8 (57.1)	5 (45.5)	20 (50.0)	0.852 ^b^
	late	8 (53.3)	6 (42.9)	6 (54.5)	20 (50.0)	

Values are presented as number (%) or mean ± standard deviation; Tests: ^a^ ANOVA, ^b^ Chi-squared test, and ^c^ Fisher’s exact test; *p* < 0.05 was considered statistically significant. Abbreviations: PASI = Psoriasis area and severity index; BMI = body mass index.

**Table 2:** The effect of vitamin D supplementation on disease severity determined by PASI score in psoriatic patients.

Disease Severity by PASI	Before Vitamin D Supplementation	After Vitamin D Supplementation	*p* Value
Mild form of disease	15 (37.5)	25 (62.5)	<0.001
Moderate form of disease	14 (35.0)	10 (25.0)	<0.001
Severe form of disease	11 (27.5)	5 (12.5)	<0.001

Data are expressed as number (%). Wilcoxon Signed Rank test; *p* < 0.05 was considered statistically significant. Abbreviation: PASI = Psoriasis area and severity index.

**Table 3:** The number and percentage of psoriatic patients with low or normal serum levels of vitamin D, homocysteine, vitamin B_12_, and folate measured before and after twelve weeks of high dose vitamin D supplementation.

Biochemical Markers		Before Vitamin D Supplementation	After Vitamin D Supplementation	*p* Value
Vitamin D				
	Low vitamin D < 75.0 ng/mL	40 (100)	8 (20.0)	0.001
	Normal vitamin D	0 (0)	32 (80.0)	
Hcy				
	Normal Hcy	15 (37.5)	33 (82.5)	0.001
	High Hcy ˃ 12.0 µmol/L	25 (62.5)	7 (17.5)	
Vitamin B_12_				
	Low vitamin B_12_ < 211.0 ng/mL	5 (12.5)	3 (7.5)	0.157
	Normal vitamin B_12_	35 (87.5)	37 (92.5)	
Folate				
	Low Folate < 3.89 µmol/L	3 (7.5)	7 (17.5)	0.102
	Normal Folate	37 (92.5)	33 (82.5)	

Data are expressed as number and percentage (%). Wilcoxon Signed Rank test; *p* < 0.05 was considered statistically significant. Abbreviation: Hcy = homocysteine.

**Table 4:** Relationship between serum Hcy and pro-inflammatory and anti-inflammatory parameters in serum of psoriatic patients before and after twelve weeks of supplementation with high dose of vitamin D.

Pro-Inflammatory and Anti-Inflammatory Parameters	Before Vitamin D Supplementation	After Vitamin D Spplementation
	r (*p*)	r (*p*)
hsCRP	−0.146 (0.374)	−0.147 (0.373)
IFN-γ	0.148 (0.370)	0.089 (0.588)
TNF-α	−0.046 (0.779)	−0.045 (0.784)
IL-1β	0.078 (0.639)	−0.338 (0.035)
IL-6	0.153 (0.354)	−0.047 (0.776)
IL-8	−0.091 (0.580)	−0.374 (0.019)
IL-17	0.266 (0.102)	0.117 (0.479)
IL-10	−0.106 (0.522)	0.046 (0.780)
IL-5	0.183 (0.264)	0.096 (0.561)

R—Pearson’s coefficient of correlation; *p* < 0.05 was considered statistically significant. The numbers of patients in both groups were 40. Abbreviation: hsCRP = high-sensitivity C-reactive protein.

## Data Availability

The article contains complete data used to support the findings of this study.
